# Identifying central behavioral and psychological symptoms associated with care time in older adults with dementia: a network analysis

**DOI:** 10.3389/fpubh.2026.1744794

**Published:** 2026-01-23

**Authors:** Chunqin Liu, Qing Luo, Ying Zhou, Huijuan Li, Haicheng Liu, Xinyang Hu

**Affiliations:** 1 Nursing Department, The Third Affiliated Hospital of Sun Yat-sen University, Guangzhou, Guangdong, China; 2 School of Nursing, Guangzhou Medical University, Guangzhou, Guangdong, China; 3 School of Health and Medicine, Guangzhou Hua Shang College, Guangzhou, Guangdong, China

**Keywords:** BPSD, care time, dementia, network analysis, older adults

## Abstract

**Background:**

While behavioral and psychological symptoms of dementia (BPSD) are generally associated with care time, the strength and nature of these associations may vary across individual symptoms.

**Aim:**

To examine these nuanced associations using a network perspective among older adults with dementia, thereby yielding more comprehensive insights.

**Design:**

A cross-sectional study design was employed.

**Methods:**

Between December 2022 and May 2023, 205 Chinese older adults with dementia were recruited from two nursing homes in Guangzhou. BPSD severity and caregiver distress were assessed using the Neuropsychiatric Inventory (NPI). The Per-Minute Care Intensity method was used to quantify the amount of formal care time received within a 24-h period. Network analysis was conducted to illustrate the complex nuanced relationships between specific BPSD symptoms and corresponding care time.

**Results:**

Network analysis identified “irritability” and “agitation/aggression” severity, along with the caregiver distress caused by these two symptoms, as central nodes in two separate networks. In addition, the severity of “aberrant motor behaviors” and “agitation/aggression,” as well as the related caregiver distress, emerged as key bridge nodes connecting BPSD symptoms with care time.

**Conclusion:**

These findings provide preliminary evidence of potential pathways through which specific BPSD symptoms are linked to care time in older adults with dementia. This underscores the importance of prioritizing targeted interventions for central and bridge symptoms such as agitation, irritability, and abnormal motor behavior. Future intervention studies are warranted to validate these findings and further refine strategies for optimizing care practice.

**Patient or public contribution:**

No patient or public contribution.

**Impact:**

A deeper understanding of the intricate connections between specific BPSD symptoms and corresponding care time will help administrators prioritize symptoms such as agitation and irritability when designing interventions aimed at reducing caregiver burden.

## 1 Background

Dementia, a progressive neurodegenerative disease characterized by cognitive decline, is a major global healthcare challenge (1). The estimated number of people with dementia worldwide was 57.4 million in 2019 and is expected to increase to 152.8 million by 2050 (2). China has the largest number of older adults with dementia, with an estimated 16.99 million current cases of Alzheimer’s and other dementias, and this number that continues to rise (3, 4). Given the increasing global disease burden of dementia, improving dementia care have become critical areas of focus.

Dementia is often accompanied by a range of behavioral and psychological symptoms of dementia (BPSD), also termed neuropsychiatric symptoms or challenging behaviors. Studies have shown that over 90% of patients with dementia experienced at least one BPSD over the course of their illness (5, 6). These symptoms include, but are not limited to, hallucinations, delusions, anxiety, depression, aggression, agitation, wandering, and socially inappropriate behaviors. BPSD symptoms are increasingly recognized as the most challenging aspects of dementia care due to their sudden onset, difficulty in treatment (7), and many negative outcomes associated with them, such as functional decline (8), accelerated disease progression (9), increased institutionalization risk, and rising healthcare costs (10). These symptoms also significantly burden caregivers, leading to reduced quality of life and distress (11), often greater than with cognitive deficits (12). The management of BPSD is further complicated by the interaction of various factors, such as unmet care needs, underlying acute medical conditions, environmental triggers, and external barriers like limited time, insufficient access to community resources, and the absence of interdisciplinary care teams. While person-centered, non-pharmacological interventions are recommended as the first-line treatment for BPSD management (13), their effectiveness remains limited (14). Caregivers and healthcare providers face difficulties in implementing targeted interventions due to insufficient evidence addressing specific symptoms. Consequently, prioritizing the identification and management of the most burdensome symptoms to caregivers is critical for developing targeted interventions and optimizing resource allocation in dementia care.

Care time, particularly the time required for BPSD management, reflects the unique care requirements of dementia patients, their dependence on caregivers, and serves as an objective measure for evaluating these needs. In this study, care time is defined as the total amount of minutes spent by formal caregivers providing long-term care services for patients over a 24-h period. A distinctive portion of this care time is allocated to BPSD management, which includes assisting with orientation, preventing dangerous events (e.g., the risk of fire or getting lost), and calming or caring for agitated or aggressive patients, among others (15). The adapted stress model proposed by Lindt et al. (16) provides a useful framework for understanding the relationship between general stressors (e.g., BPSD symptoms) and variables reflecting objective caregiving burden (i.e., care time). Specifically, these stressors can influence the caregiver’s primary appraisal of their severity, which in turn affects the amount of caregiving time required (17). Previous studies have reported that greater use of care time for BPSD management is associated with higher prevalence and severity of neuropsychiatric symptoms (18, 19). According to resource allocation theory (20), caregivers need to allocate scarce resources, such as time, among various care demands to achieve a balance. Consequently, identifying key BPSD symptoms is crucial for the efficiently allocation of caregiving time resources.

However, existing evidence on which specific BPSD symptoms are most strongly associated with care time remains limited and inconsistent (19, 21). Although nearly all symptoms may contribute to care time use, individual neuropsychiatric symptoms are likely to exhibit differential associations with care time, reflecting the heterogeneity, frequent comorbidity, and mutual reinforcement inherent in BPSD (22, 23). For example, Vislapuu et al. (21) found that the agitation cluster (i.e., agitation, disinhibition, irritability, and aberrant motor behaviors) was positively associated with increased direct formal care time, whereas mood and psychosis clusters were not. In contrast, Wu (24) reported that, at the individual symptoms level, only irritability in dementia was associated with increased BPSD-related care time. Similarly, Chahine et al. (25) found that anxiety severity exerted the largest effect size on time dependency among patients with Parkinson’s disease. Despite these insights, prior research has largely examined the relationship between BPSD and care time either at the cluster level or by considering isolated symptoms, which may overlook the complex co-occurrence and interrelationships among symptoms that are relevant to caregiving demands. Consequently, the extent to which individual BPSD symptoms jointly relate to care time remains insufficiently understood. Network analysis, by contrast, is an inherently exploratory method that provides a comprehensive view of relational patterns (26). It is an appropriate statistical approach for understanding complex phenomena, as it requires acknowledging the nonlinear and dynamic nature of cause and effect (27). Therefore, the aim of this study is to examine the network structure of BPSD and corresponding care time, with a focus on identifying the central and bridging symptoms. We anticipate that the finding of this research may offer new insights into personalized BPSD management and contribute to the more efficient allocation of care time, thereby enhancing the effectiveness of caregiving practice in dementia.

## 2 The study

### 2.1 Aim

To analyze the nuanced associations between BPSD and its associated care time using network analysis among Chinese older adults with dementia.

## 3 Methods

### 3.1 Study design

A cross-sectional study design was employed.

### 3.2 Participants and recruitment

A convenience sampling was conducted in two nursing homes in Guangzhou from December 2022 to May 2023. We collected the data through the care staff who generally cared for or were close to the participants completed the questionnaires while referencing medical and care records. Inclusion criteria for older adults with dementia include (a) aged 60 years and older, expected survival 6 months or more as judged by the responsible physician, (b) having a clinical diagnosis of dementia, regardless of etiology, (c) residing in the nursing homes for at least 1 month, and (d) participants or their legal guardians provided informed consent with this study. Participants were excluded if they have a history of psychiatric disorders, or with an acute or end-stage medical condition. Eligible caregivers were nursing assistants mainly responsible for daily care, who was 20 years and older, had possessed a Vocational Certificate, were working longer than 6 months in dementia care center, cognitively intact, and voluntarily consented to participate in this study, whereas nursing assistants who trainees were excluded. The sample size was calculated following the guidelines in Epskamp and Fried’s tutorial (28), which suggest a minimum of P(P − 1)/2 participants to ensure sufficient statistical power for a partial correlation network with P nodes. In this study, P represented 12 BPSD symptoms and 1 BPSD caregiving time (nodes), indicating a minimum requirement of 13 × 12/2 = 78. Initially, 230 participants were recruited, with a final sample of 205 patients, yielding a participation rate of 89.13%.

### 3.3 Data collection

Data collection was conducted by a trained research team comprising two doctoral nursing students and two master’s nursing students. Prior to formal data collection, all surveyors completed a three-day training program and spent 1 week residing in the nursing home to familiarize themselves with caregiving routines and minimize the risk of missing care activities. Two data collection methods were used: (1) direct, one-on-one real-time observation and time recording of caregiving activities; and (2) in-person interviews with caregivers to collect contextual information related to care delivery. Care time was measured using the Per-Minute Care Intensity Method. Trained investigators conducted one-on-one shadowing of each caregiver over a continuous 24-h period (day shift: 06:00–18:00; night shift: 18:00–06:00) and recorded all care activities in real time with standardized electronic stopwatches (Isport Multi-Function Timer; accuracy to the second). Time calculation followed two predefined rules: (1) when a caregiver provided care to multiple residents simultaneously, the recorded duration was equally distributed across recipients; and (2) when multiple caregivers jointly cared for one resident, the duration was multiplied by the number of caregivers.

### 3.4 Measures

#### 3.4.1 Participants characteristics

Patients characteristics included age, sex, education, length of residence in the nursing home, number of chronic diseases, number of medications taken, and dementia subtypes. Specifically, the number of chronic diseases was defined as the total count of diagnosed conditions based on the International Classification of Diseases, 10th Edition (ICD-10). Dementia subtypes were classified as Alzheimer’s dementia (AD), vascular dementia, and other dementia subtypes.

#### 3.4.2 Measurement of care time

The care time measurement questionnaire was developed by Zhang (29) based on *the Guangzhou Long-Term Care Insurance Basic and Medical Care Service Projects (Sui Yi Bao Gui Zi [2020] No. 10)* (30). It provides an objective method for assessing care needs among older adults with dementia. Care time consisted of the following parts: (1) direct basic care time, referring to the time required to assist with fundamental activities of daily living (e.g., feeding, personal hygiene, transferring, toileting, and bathing); (2) indirect care time, which is the time required to help domestic tasks (e.g., doing laundry and cleaning); (3) medical assistance time, involving activities such as wound care and injection administration; and (4) the time spent for managing the BPSD or supervision (e.g., helping with orientation, preventing dangerous situations, and calming or caring for agitated or aggressive patients). Previous studies have shown that the majority of care time in long-term care settings is devoted to direct care, and that increases in care need levels are primarily reflected by increases in time spent on direct care activities, whereas other care components show relatively smaller variation across care need levels (31, 32). Accordingly, during data collection, all caregiving activities except indirect care were continuously observed and recorded. In the current study, analyses focused on BPSD-related care time, which was operationalized as the time spent on caregiving activities directly attributable to behavioral and psychological symptoms of dementia, beyond basic or medical care. Care time was recorded in minutes per day.

#### 3.4.3 Mini-mental state examination (MMSE)

The MMSE was used to assess dementia severity (33). The total score ranges from 0 to 30, with higher scores indicating better cognitive functioning. In the current study, participants were stratified according to MMSE scores based on the UK clinical guidelines as follows: mild dementia (MMSE score: 21–26), moderate dementia (MMSE 15–20), moderately severe dementia (MMSE 10–14), and severe dementia (MMSE <10) (34).

#### 3.4.4 Neuropsychiatric inventory questionnaire (NPI-Q)

The NPI-Q was used to assess neuropsychiatric symptoms in dementia patients over the last month (35). It consists of 12 domains: delusions, hallucinations, agitation/aggression, depression, anxiety, euphoria, apathy, disinhibition, irritability, aberrant motor behaviors, night-time disturbances, and appetite/eating disturbances. Each symptom was first rated as “yes,” “no,” or “inapplicable.” For endorsed symptoms, severity was assessed on a 4-point scale (0–3), frequency on a 4-point scale (1–4). and care burden was rated on a 6-point scale (0–5). Symptoms that were not present were assigned a severity score of 0, and care burden was evaluated only when symptoms were present. Total severity scores were calculated by multiplying severity and frequency scores, ranging from 0 to 144. Total care burden scores ranged from 0 to 60, with higher scores indicating greater symptom severity or caregiver burden.

### 3.5 Ethical considerations

This study was approved by the Institutional Review Board of Guangzhou Medical University (Ethical approval number: L202307006). Throughout the study, we adhered to the principles outlined in the Declaration of Helsinki, ensuring participants’ autonomy and confidentiality were fully respected. Informed consent was obtained from all participants, or their legal guardians, or the facility directors, prior to the commencement of the survey.

### 3.6 Statistical analysis

All analyses were conducted using R version 4.3.1. First, descriptive statistics were performed to summarize participants’ demographic characteristics and study variables. Care time (minutes) and BPSD symptom scores were assessed for normality using the Shapiro-Wilks test and were found to be non-normally distributed (*p* < 0.05). consequently, non-parametric tests, including the Mann–Whitney U test and Kruskal-Wallis test, were used to examine differences in BPSD-related care time across demographic groups. Prior to network estimation, all variables were transformed using a nonparanormal (npn) method to address deviations from multivariate normality (36). Spearman correlations analysis showed that most BPSD symptoms were correlated with care time (*r* = 0.15–0.44, *p* < 0.05), and multicollinearity diagnostics indicated no violations (all variance inflation factors < 3; tolerance > 0.10), supporting the appropriateness of network modeling. Subsequently, a Gaussian Graphical Model (GGM) was estimated using the *qgraph* package to examine conditional associations between BPSD symptoms and care time (37). Network estimation employed LASSO regularization with the Extended Bayesian Information Criterion (EBICglasso) to obtain a sparse and clinically interpretable structure (38), with the tuning parameter set at 0.50 (39). In the resulting undirected network, nodes represent individual BPSD symptom and care time, while edges reflect partial correlation coefficients between nodes after controlling for all others. Edges thickness indicates the strength of the associations among nodes, and green edges refers to positive correlations while the red edges, to negative ones (28). The network layout was generated using the *Fruchterman-Reingold* algorithm, which places stronger associations centrally and weaker ones toward the periphery (40).

Then, centrality indices were computed to assess the relative importance of each node, including betweenness, closeness, strength, and expected influence (EI). Among these, EI was the primary centrality metric reported in the current study, as it has shown greatest reliability and interpretability, especially in networks involving both positive and negative associations (41). Strength was not reported because it does not capture negative associations (41). Betweenness and closeness were also excluded due to their limited stability and robustness in networks (42). To assess bridge centrality, we calculated the bridge expected influence (BEI) of each node using the “*networktools*” package (43). To assess the accuracy of edge weights in node pairs, we conducted a non-parametric bootstrap procedure with 1,000 bootstrapped samples using the “*bootne*t” package. A narrow 95% confidence interval (CI) indicates a more accuracy network. We also calculated the correlation stability coefficient (CS) to evaluate the robustness of centrality metrics. According to Epskamp’s study (37), a CS coefficient above 0.25 is considered acceptable for stable interpretation.

## 4 Results

### 4.1 Participants characteristics and differences in care time

A total of 205 older adults with dementia residing in nursing homes were included in the analysis. The average age was 85.35 years (SD = 7.67), ranging from 63 to 105 years. Of them, 72.68% were females, 57.07% had an educational level of elementary school or below. Nearly half (45.85%) had lived in the nursing homes for more than 3 years, 91.22% reported two or more chronic diseases, 53.20% were prescribed five or more medications. Alzheimer’s disease was the most common diagnosis (84.04%). The mean MMSE score was 7.13 ± 7.74, with 10.20% classified as mild dementia,12.70% as moderate, 16.6% as moderately severe, and 60.50% as severe dementia. The median BPSD-related care time was 2.72 min per day (interquartile range: 0.00–6.27). The BPSD-related care time did not differ by age, gender, education, length of residence, medication, or type of dementia (*p* > 0.05 for all scores), while significant differences were observed across dementia severity groups (*p* < 0.05), as shown in Table 1.

**Table 1:** Analysis of general characteristics associated with BPSD-related care time (*n* = 205).

Variables	*n* (%)/mean (SD)	Median (IQR)	*p*
Age	85.35 (7.67)		0.103^a^
Sex			
Female	56 (27.32)	3.54 (0, 5.48)	0.885^b^
Male	149 (72.68)	2.63 (0, 6.70)	
Education			
Elementary school and under	117 (57.07)	3.57 (0, 7.00)	0.757^c^
Middle school	36 (17.56)	2.09 (0, 5.48)	
High school	34 (16.59)	2.24 (0, 5.79)	
College and above	19 (9.27)	2.43 (0, 6.03)	
Length of residence (years)			
<1	46 (22.44)	2.46 (0, 6.18)	0.975^c^
1–3	65 (31.71)	3.00 (0, 6.06)	
>3	56 (27.32)	2.69 (0, 6.78)	
Number of chronic diseases			
0–1	18 (8.78)	4.72 (2.10, 9.61)	0.061^b^
≥2	187 (91.22)	2.63 (0, 6.17)	
Medication			
<5	96 (46.80)	2.63 (0, 6.48)	0.698^b^
≥5	109 (53.20)	3.17 (0, 6.27)	
Type of dementia			
Alzheimer’s disease	182 (84.40)	4.10 (0.57, 6.52)	0.615^c^
Vascular dementia	20 (9.80)	2.58 (0, 6.27)	
Other types	12 (5.90)	4.02 (0, 7.54)	
Dementia severity			
Mild	21 (10.24)	1.45 (0, 3.50)	0.014^c^
Moderate	26 (12.68)	1.90 (0, 4.57)	
Moderately severe	34 (16.59)	1.52 (0, 4.12)	
Severe	124 (60.49)	4.28 (0.19, 7.28)	

Medication represents the number of drug types taken daily, ^a^Spearman correlation test, ^b^nonparametric Mann–Whitney U-test, ^c^nonparametric Kruskal-Wallis test.

### 4.2 Descriptive statistics for BPSD symptoms and BPSD-related care time

Table 2 presents abbreviation, scale items, expected influence and bridge expected influence (raw-scores) of BPSD symptoms and BPSD-related care time.

**Table 2:** Abbreviation and expected influence for items of BPSD symptoms and BPSD-related care time (*n* = 205).

Variables	Item	Category	Abbreviation	EI	BEI
NPI1	Delusions	Severity	NPI1-1	0.41	0.00
		Distress	NPI1-2	0.38	0.00
NPI2	Hallucinations	Severity	NPI2-1	0.38	0.06
		Distress	NPI2-2	0.55	0.08
NPI3	Agitation/Aggression	Severity	NPI3-1	0.71	0.23
		Distress	NPI3-2	0.96	0.21
NPI4	Depression	Severity	NPI4-1	0.26	0.00
		Distress	NPI4-2	0.58	0.00
NPI5	Anxiety	Severity	NPI5-1	−0.05	0.00
		Distress	NPI5-2	−0.41	0.00
NPI6	Euphoria	Severity	NPI6-1	−0.03	0.00
		Distress	NPI6-2	0.18	0.00
NPI7	Apathy	Severity	NPI7-1	−0.17	−0.05
		Distress	NPI7-2	0.01	0.00
NPI8	Disinhibition	Severity	NPI8-1	−0.09	0.00
		Distress	NPI8-2	−0.18	0.02
NPI9	Irritability	Severity	NPI9-1	0.72	0.08
		Distress	NPI9-2	0.99	0.09
NPI10	Aberrant motor behaviors	Severity	NPI10-1	0.46	0.20
		Distress	NPI10-2	0.52	0.21
NPI11	Night-time disturbance	Severity	NPI11-1	0.55	0.11
		Distress	NPI11-2	0.69	0.16
NPI12	Appetite/eating disturbances	Severity	NPI12-1	0.05	0.00
		Distress	NPI12-2	0.26	0.00
BPSD-related care time				0.62	0.62
				0.77	0.77

EI, Expected Influence (raw-scores); BEI, Bridge Expected Influence (raw-scores).

### 4.3 Network structure

#### 4.3.1 The BPSD severity-care time network

**Figure 1:**
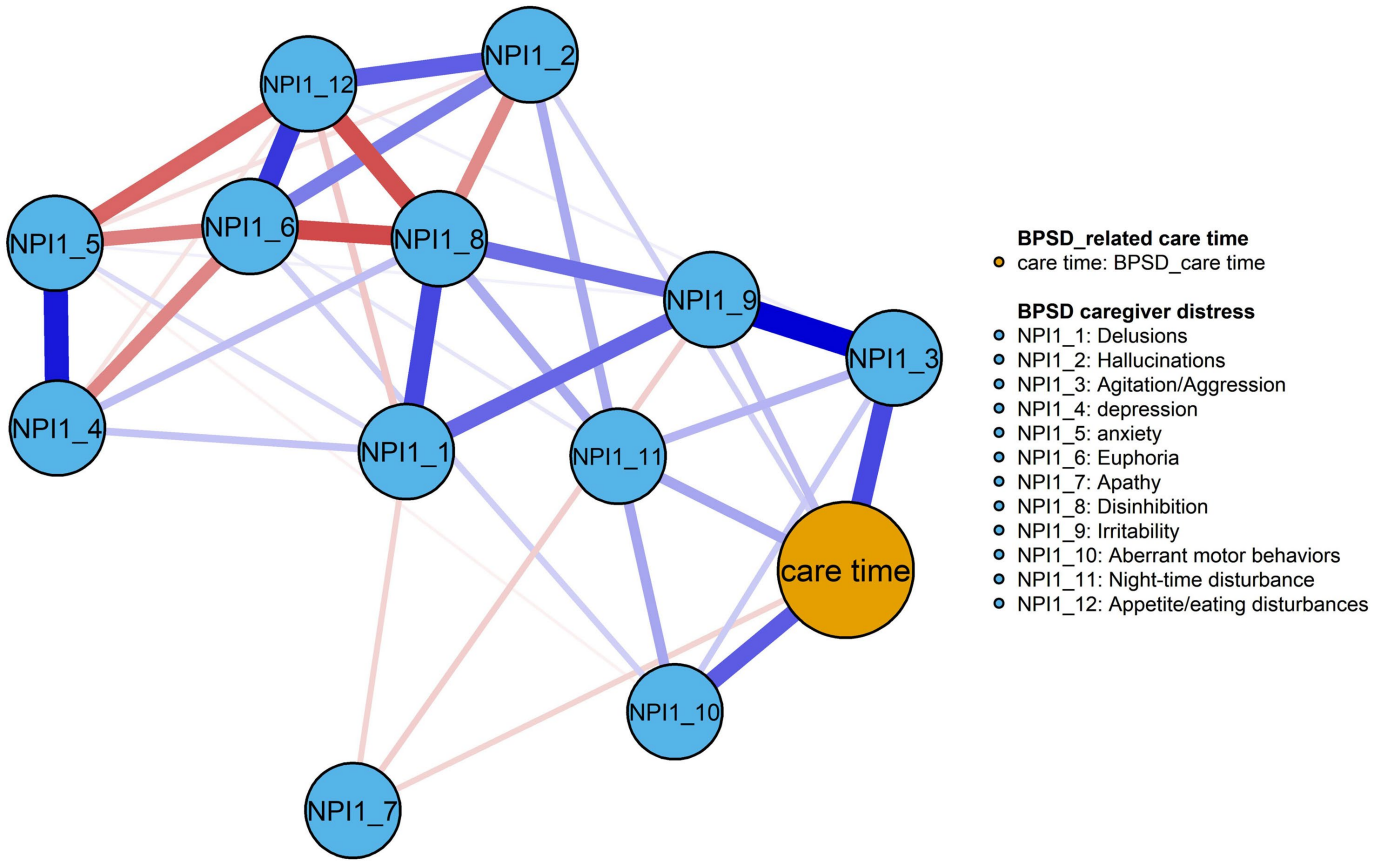
The network model of the BPSD severity and its associated care time in older adults with dementia (*n* = 205). Blue line = positive correlation; red line = negative correlation; thickness of line represents the strength of edge.

**Figure 2:**
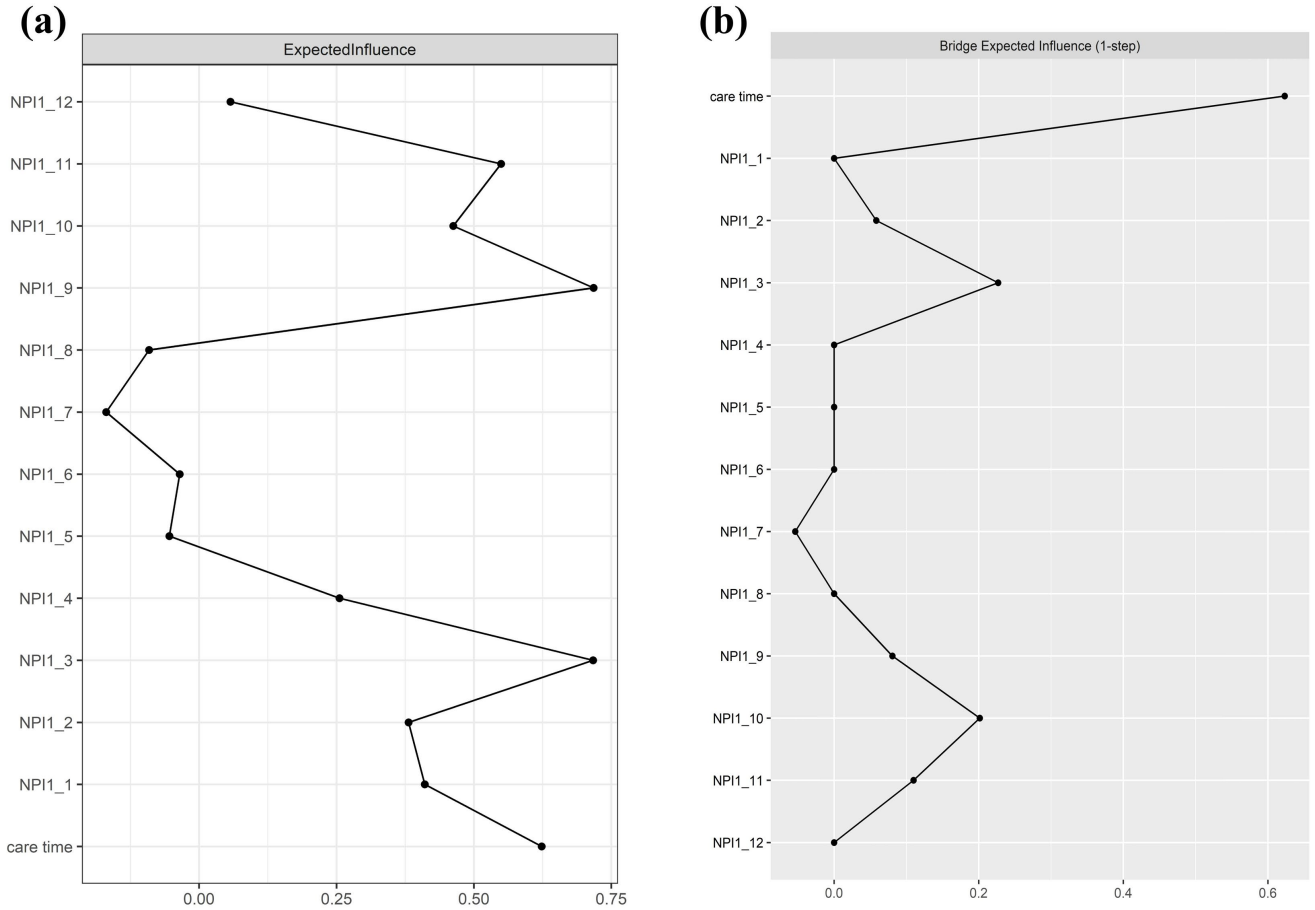
Centrality **(a)** and bridge centrality (b) in the BPSD severity and care time network (raw-scores).

All BPSD symptoms and care time variables were residualized on dementia severity prior to network estimation, ensuring that edges reflect associations independent of severity. Figure 1 shows the resulting network, comprising 39 non-zero edges, with edge weights ranging from −0.22 to 0.31. Among them, the model contains more positive edges (*n* = 26) than negative edges (*n* = 13), see Supplementary Table S1. BPSD-related care time was most strongly associated with NPI1_3 and NPI1_10, with edge weights of 0.23 and 0.20, respectively, while other BPSD severity showed weaker or negligible correlations with its associated care time. Regarding node centrality, NPI1_9 exhibited the highest centralities within the network (EI = 0.72), followed by NPI-3 (EI = 0.71), see Table 2 and Figure 2a. Within the BPSD cluster, NPI1_3 had the highest bridge strength influence, followed by NPI1_10 (see Figure 2b). Furthermore, the bootstrapped 95% confidence interval (CI) for edge weights in the BPSD severity-care time network was narrow, indicating that the estimation of edge weights was accurate (Supplementary Figure S1). Node EI yielded a CS-coefficient of 0.44, indicating the stability of the network (Supplementary Figure S2). Non-parametric bootstrap difference tests (Supplementary Figures S3, S4) revealed that the strongest edges and central nodes differed significantly from others, supporting the robustness of the observed network structure.

#### 4.3.2 The BPSD distress-care time network

**Figure 3:**
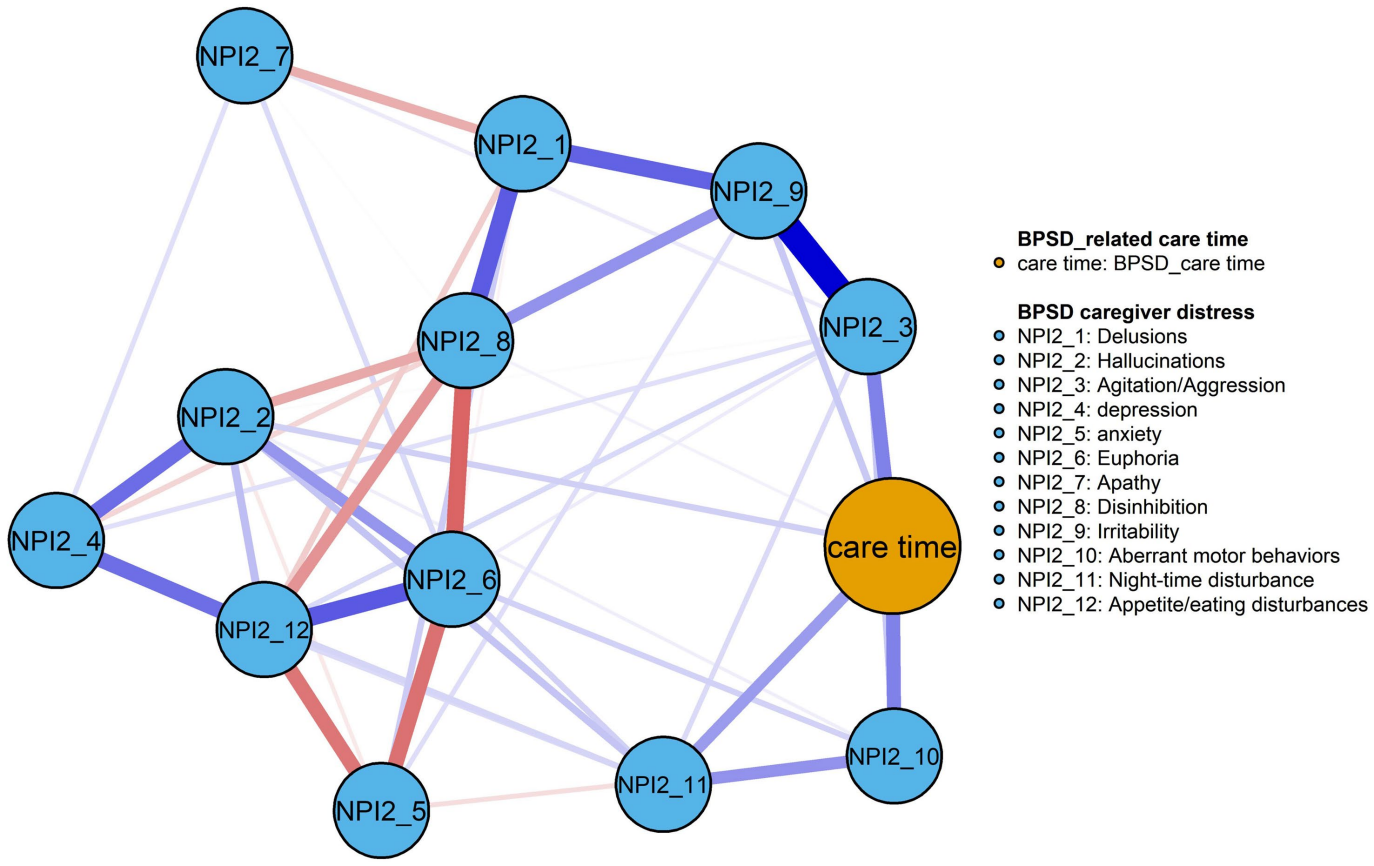
The network model of the BPSD-related caregiver distress and its associated care time in older adults with dementia (*n* = 205). Blue line = positive correlation; red line = negative correlation; thickness of line represents the strength of edge.

**Figure 4:**
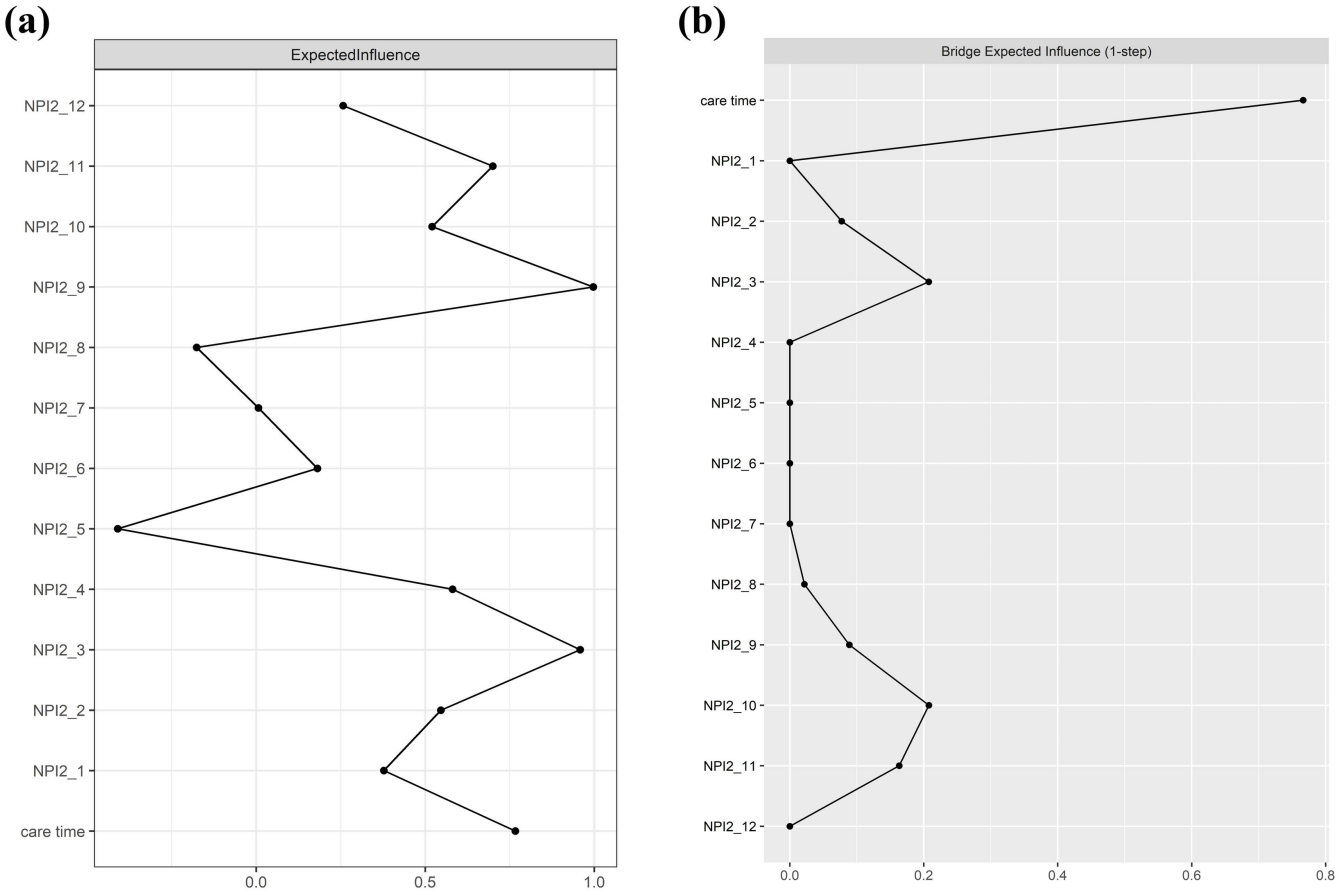
Centrality **(a)** and bridge centrality **(b)** in the BPSD distress and care time network (raw-scores).

Figure 3 presents the BPSD-related caregiver distress-care time network after adjusting for dementia severity. The network comprises 43 non-zero edges, with edge weights ranging from −0.22 to 0.42. Most edges were positive (32 positive; 11 negative), see Supplementary Table S2. BPSD-related care time showed strongest and equal associations with NPI2_10 and NPI2_3 (edge weights = 0.21), whereas the remaining symptoms distress exhibited weak or negligible associations, suggesting a comparatively limited contribution to care burden. Regarding node centrality, NPI2_9 demonstrated the highest Expected Influence (EI = 0.99), followed by NPI2_3 (EI = 0.96), indicating their dominant roles within the network (Table 2; Figure 4a). Within the BPSD cluster, NPI2_10 exhibited the highest bridge strength influence, followed by NPI2_3 (see Figure 4b). The narrow bootstrapped 95% confidence intervals supported adequate accuracy of edge weight estimation (Supplementary Figure S5). EI centrality demonstrated a CS-coefficient of 0.52, indicating moderate stability (Supplementary Figure S6). Non-parametric bootstrap difference tests (Supplementary Figures S7, S8) revealed that the strongest edges and central nodes differed significantly from others, supporting the robustness of the observed network structure.

## 5 Discussion

This study is the first to examine the association between BPSD and the corresponding care time using a network approach in a sample of older adults with dementia, offering specific insights into their complex interrelationship. The findings identified that the severity “irritability” and “agitation/aggression,” along with the caregiver distress caused by these two symptoms, as central nodes within two distinct networks. In addition, “agitation/aggression” and “aberrant motor behaviors” emerged as key bridge nodes linking BPSD symptoms with the associated care time.

Our findings identified the severity of “irritability” and “agitation/aggression,” along with caregiver distress related to these two symptoms, emerged as central nodes for older adults with dementia in two separate network structures. Notably, both the severity of “agitation/aggression” and the associated caregiver distress were closely connected to care time. The high centrality of “irritability” is aligned with previous cross-sectional data indicating that irritability independently contributes to care time in community-dwelling dementia patients (24). Similarly, a retrospective analysis of dementia patients in US showed that the presence of agitation was associated with longer caregiving time, whether provided by professional or non-professional, and higher healthcare costs (44). Specifically, dementia patients exhibiting irritability or agitation toward their caregivers often demonstrate abusive word and resistance to care, which may exacerbate the difficulty of caregiving activities and consequently extend the time required for management (45). Furthermore, our results, to some extent, support the adapted stress model, suggesting that exposure to those distressing neuropsychiatric symptoms can directly impact care time allocation, an indicator of objective caregiver burden. Additionally, a network analysis of 9,691 older adults with Alzheimer’s disease identified agitation/aggression as the central symptom within the neuropsychiatric symptoms, which likely increases the likelihood of other additional symptoms being present (46). From a network perspective, targeted interventions that prevent or alleviate such core symptoms (e.g., Person-centered care) (47), or support caregivers in managing specific challenging behaviors (e.g., Aggression Prevention Training) (48), may not only improve overall symptomatology but also reduce care time and healthcare costs. Additionally, our findings suggest caregiver distress caused by these problematic behaviors as an important target for intervention when designing support programs.

Within the BPSD communities, “agitation/aggression” and “aberrant motor behaviors” severity, along with caregiver distress caused by these two symptoms, were identified as key bridge nodes linking BPSD symptoms to the time spent on BPSD management. These bridge nodes exhibited the strongest connections to care time across the network models (edge weights = 0.20–0.23), indicating that they contribute disproportionately to caregiving demands, independent of other symptoms. These findings align with previous research reporting that agitation/aggression and aberrant motor behaviors are among the most distressing and time-consuming symptoms for caregivers (49). For instance, higher levels of agitation were found to be associated with increased direct care time in nursing home residents with dementia (21), and the presence of agitation and aberrant motor behaviors independently contributed to greater supervisory care time in a national sample of older adults with dementia (50). Aggression and wandering (a subtype of aberrant motor behaviors) are also among the most distressing behaviors for caregivers (45) and are correlated with time spent providing care (51). This may be due to their links functional limitations and increased risk of falls, self-harm, and other unsafe behaviors (52). From a network perspective, bridge symptoms facilitate the transmission of effects across communities, representing pathways through which specific BPSD symptoms influence care allocation. Clinically, these findings suggest that targeting agitation/aggression and aberrant motor behaviors may be effective strategy to optimize care time and reduce caregiver burden.

### 5.1 Limitations and strengths

This study has several limitations. First, the network structures examining the relationships between BPSD severity and the corresponding care time, as well as between BPSD-related caregiver stress and the corresponding care time, were derived from cross-sectional data. This design limits causal inference and the ability to draw the actual effects of interventions. Future studies employing longitudinal or intervention designs are needed to examine the temporal dynamics of the BPSD-care time network and the efficacy of targeted interventions over time. Second, care time was measured in long-term care facilities under routine care conditions using a predefined classification of caregiving activities. Under this approach, some overlap between BPSD-related care time and other care components is unavoidable in real-world caregiving. Care time estimates may also vary across observation periods due to contextual factors, such as seasonal conditions or differences in caregiving practices among care staff. Future studies using repeated measurements and digital tracking tools may improve assessment accuracy. Third, the identified network structure was derived from a sample of older nursing home residents in Guangzhou, which may not be representative of the broader older adult population in China. This limits the generalizability of the findings to other regions or care contexts. Future studies with larger and more diverse samples, including community-dwelling older adults and participants from multiple geographical areas, are needed to enhance external validity. Finally, the presence and severity of BPSD symptoms, as well as caregiver distress, were assessed based on caregiver self-report, which may be subject to reporting bias. This limitation should be considered when interpreting the study findings. Despite these limitations, this study has several strengths. The joint assessment of symptom severity and caregiver distress allows for a more comprehensive characterization of BPSD-related caregiving burden. In addition, the use of real-world data from institutional care settings enhances the ecological validity of the findings and supports their relevance for informing targeted interventions and individualized care planning.

## 6 Conclusion

In summary, this study examined the associations between BPSD symptoms and corresponding care time from a network analysis perspective among older adults with dementia. The findings identified irritability, aberrant motor behaviors, and agitation/aggression as central symptoms, suggesting that they may be important targets for intervention. Prioritizing the management of these symptoms may help inform more efficient allocation of care resources and time and potentially alleviate caregiver burden in institutional care settings. Future longitudinal and intervention studies are warranted to validate these findings and to further refine strategies for addressing the complex relationships between BPSD symptoms and caregiving time.

## Data Availability

The datasets used and/or analyzed during the current study are available from the corresponding author on reasonable request.
