# Supplementary material for: Identifying central behavioral and psychological symptoms associated with care time in older adults with dementia: a network analysis
Source: Front Public Health. 2026 Jan 23;14:1744794. doi: 10.3389/fpubh.2026.1744794 (PMC12875950; doi:10.3389/fpubh.2026.1744794)
Supplement: Supplementary file 1 [file Data_Sheet_1.docx]

**Supplementary Materials**

1. Table S1. All edges weights within the BPSD-related severity and care time network

2. Figure S1. Accuracy of edge weights in the BPSD-related severity and care time network

3. Figure S2. The stability of centrality index-expected influence using case dropping bootstrap in the BPSD-related severity and care time network.

4. Figure S3. Bootstrapped difference test for edge weights in the BPSD-related severity and care time network

5. Figure S4. Bootstrapped difference test for expected Influence in the BPSD-related severity and care time network

6. Table S2. All edges weights within the BPSD-related distress and care time network

7. Figure S5. Accuracy of edge weights in the BPSD-related distress and care time network

8. Figure S6. The stability of centrality index-expected influence using case dropping bootstrap in the BPSD-related distress and care time network.

9. Figure S7. Bootstrapped difference test for edge weights in the BPSD-related distress and care time network

10. Figure S8. Bootstrapped difference test for expected Influence in the BPSD-related distress and care time network

Table S1. All edges weights within the BPSD-related severity and care time network

| Variables | care time | NPI1_1 | NPI1_2 | NPI1_3 | NPI1_4 | NPI1_5 | NPI1_6 | NPI1_7 | NPI1_8 | NPI1_9 | NPI1_10 | NPI1_11 | NPI1_12 |
| --- | --- | --- | --- | --- | --- | --- | --- | --- | --- | --- | --- | --- | --- |
| care time | 0.00 |  |  |  |  |  |  |  |  |  |  |  |  |
| NPI1_1 | 0.00 | 0.00 |  |  |  |  |  |  |  |  |  |  |  |
| NPI1_2 | 0.06 | 0.00 | 0.00 |  |  |  |  |  |  |  |  |  |  |
| NPI1_3 | 0.23 | 0.00 | 0.00 | 0.00 |  |  |  |  |  |  |  |  |  |
| NPI1_4 | 0.00 | 0.07 | 0.00 | 0.00 | 0.00 |  |  |  |  |  |  |  |  |
| NPI1_5 | 0.00 | 0.04 | -0.04 | 0.00 | 0.29 | 0.00 |  |  |  |  |  |  |  |
| NPI1_6 | 0.00 | 0.00 | 0.16 | 0.00 | -0.15 | -0.16 | 0.00 |  |  |  |  |  |  |
| NPI1_7 | -0.05 | -0.05 | 0.00 | 0.00 | 0.00 | 0.00 | 0.00 | 0.00 |  |  |  |  |  |
| NPI1_8 | 0.00 | 0.23 | -0.14 | 0.00 | 0.08 | 0.00 | -0.22 | 0.00 | 0.00 |  |  |  |  |
| NPI1_9 | 0.08 | 0.19 | 0.00 | 0.31 | 0.00 | 0.02 | 0.00 | -0.06 | 0.18 | 0.00 |  |  |  |
| NPI1_10 | 0.20 | 0.00 | 0.04 | 0.07 | 0.00 | -0.02 | 0.06 | 0.00 | 0.00 | 0.00 | 0.00 |  |  |
| NPI1_11 | 0.11 | 0.00 | 0.10 | 0.09 | 0.00 | 0.00 | 0.03 | 0.00 | 0.00 | 0.00 | 0.11 | 0.00 |  |
| NPI1_12 | 0.00 | -0.07 | 0.20 | 0.02 | -0.03 | -0.19 | 0.25 | 0.00 | -0.22 | 0.00 | 0.00 | 0.10 | 0.00 |


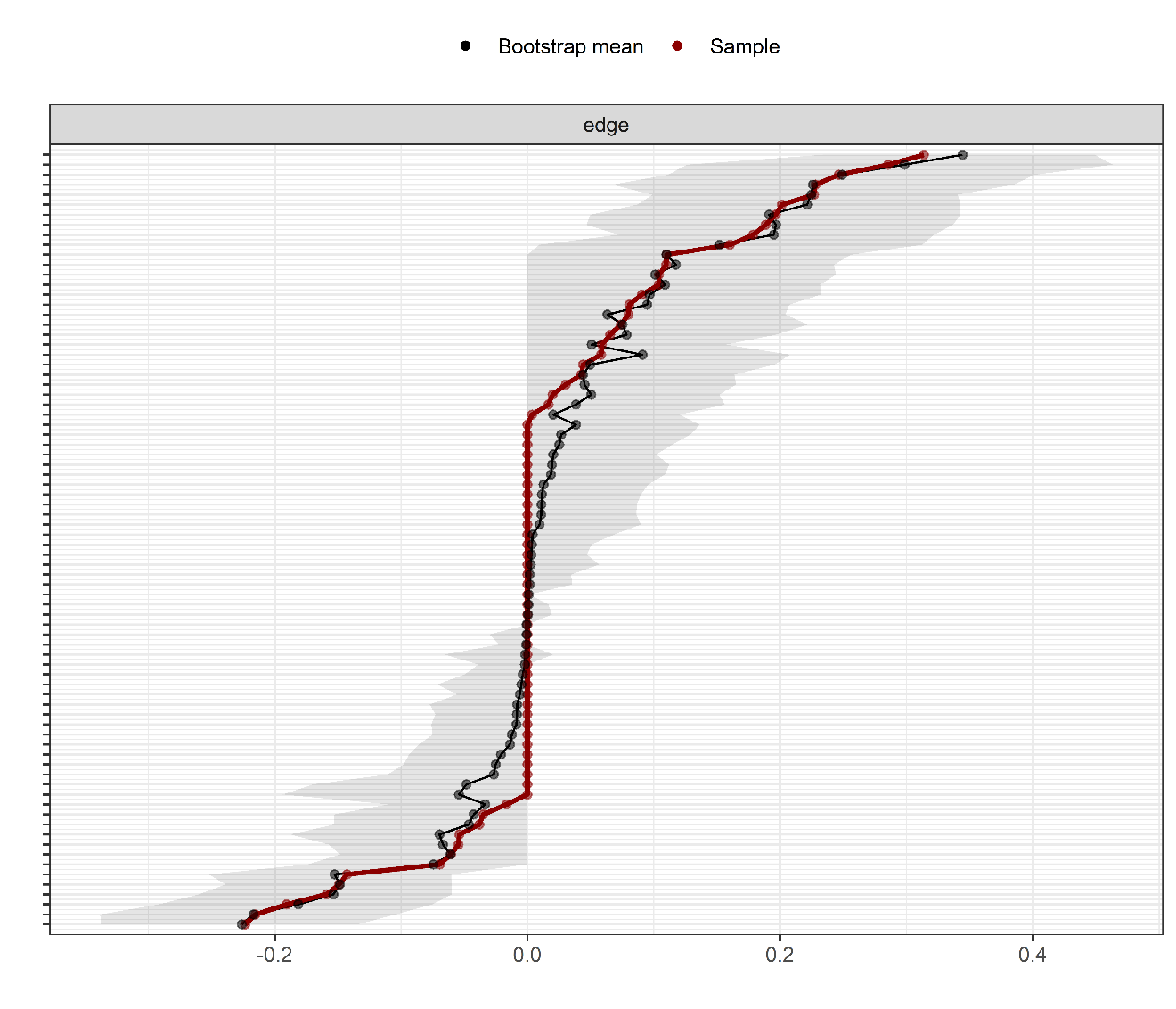


Figure S1. Accuracy of edge weights in the BPSD-related severity and care time network


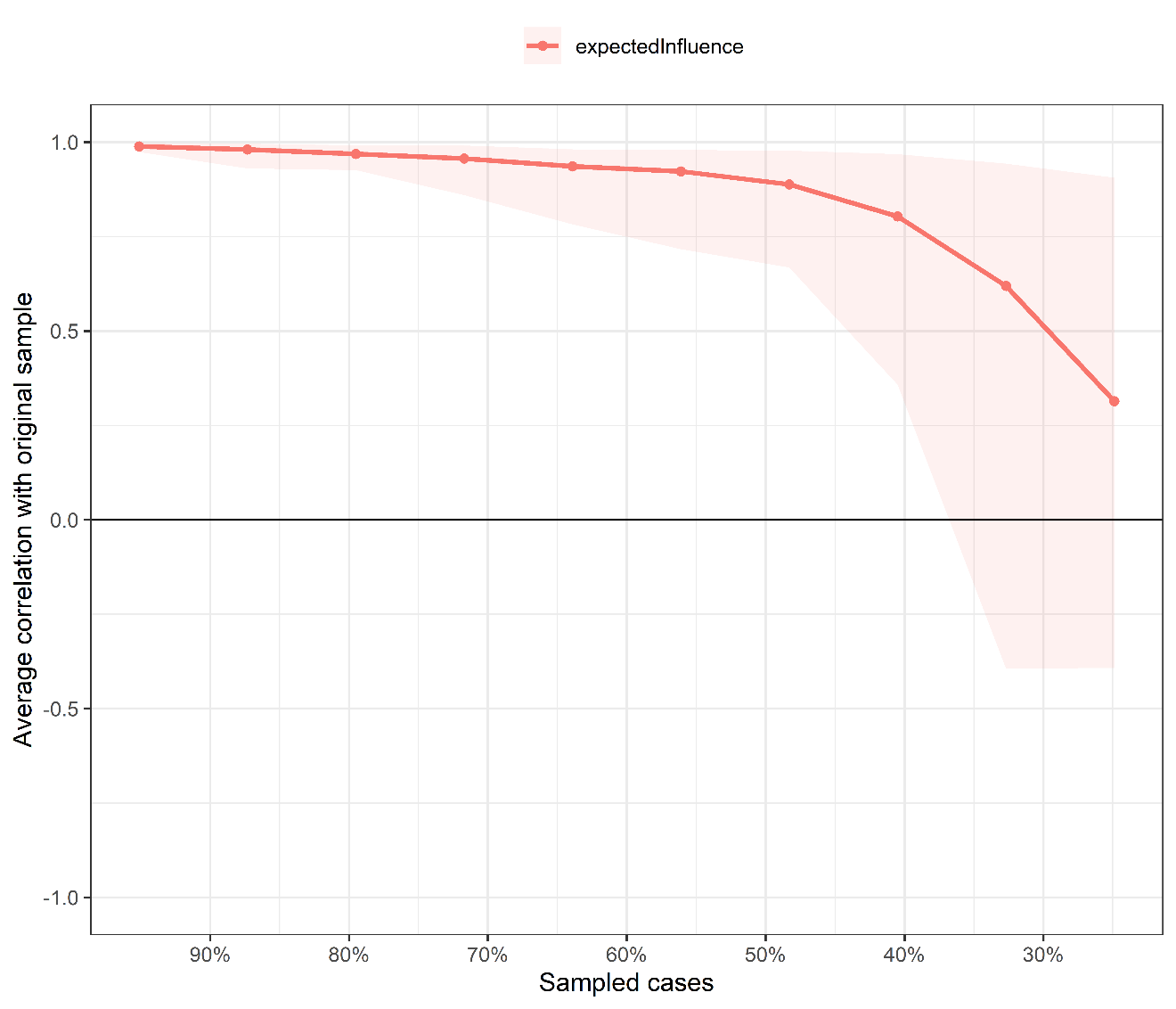


Figure S2. The stability of centrality index-expected influence using case dropping bootstrap in the BPSD-related severity and care time network.


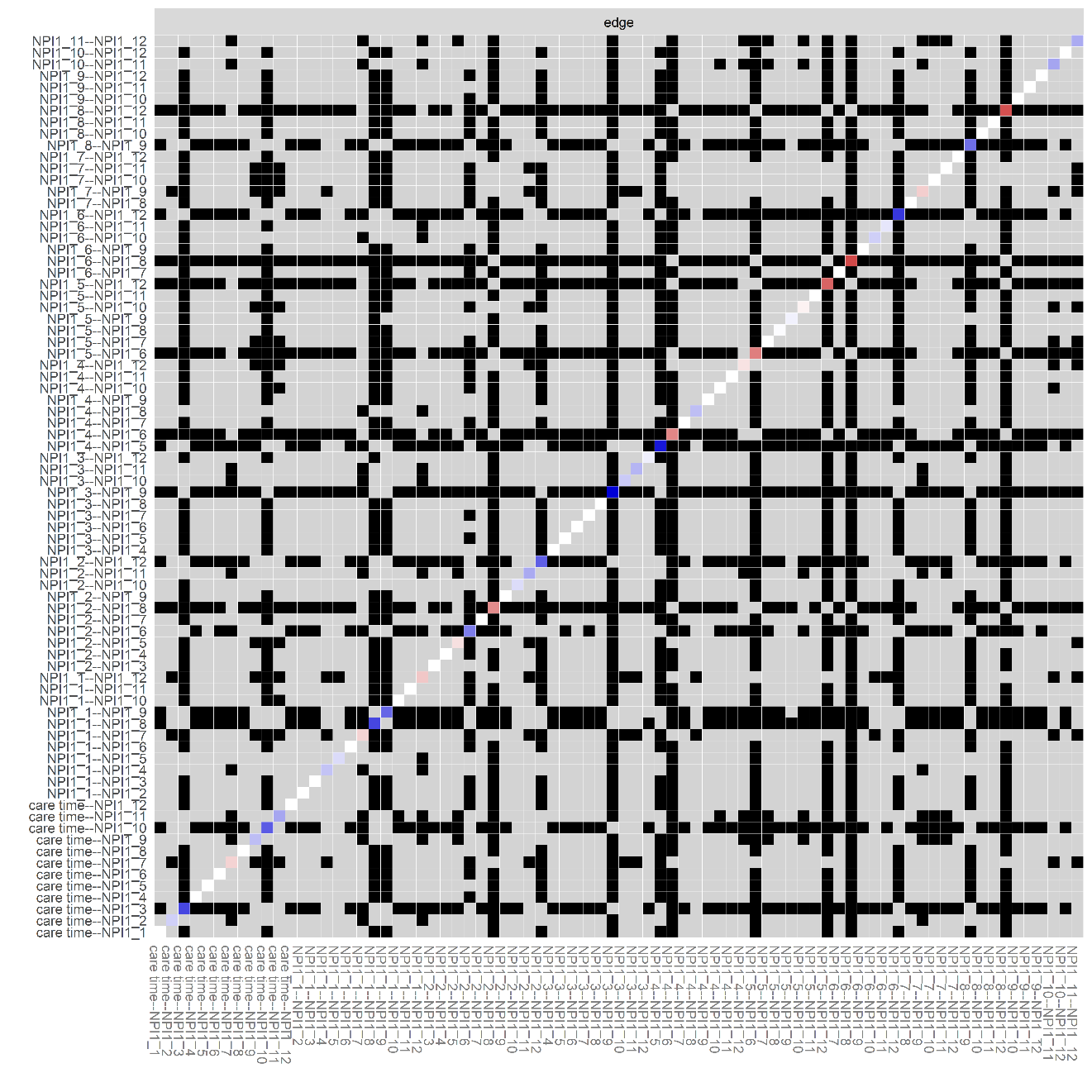


Figure S3. Bootstrapped difference test for edge weights in the BPSD-related severity and care time network

*Note*: Gray boxes indicate edge weights that do not differ significantly from one another, while black boxes indicate edge weights that do differ significantly. Blue and red boxes on the diagonal correspond to edge weights with positive and negative correlations, respectively.


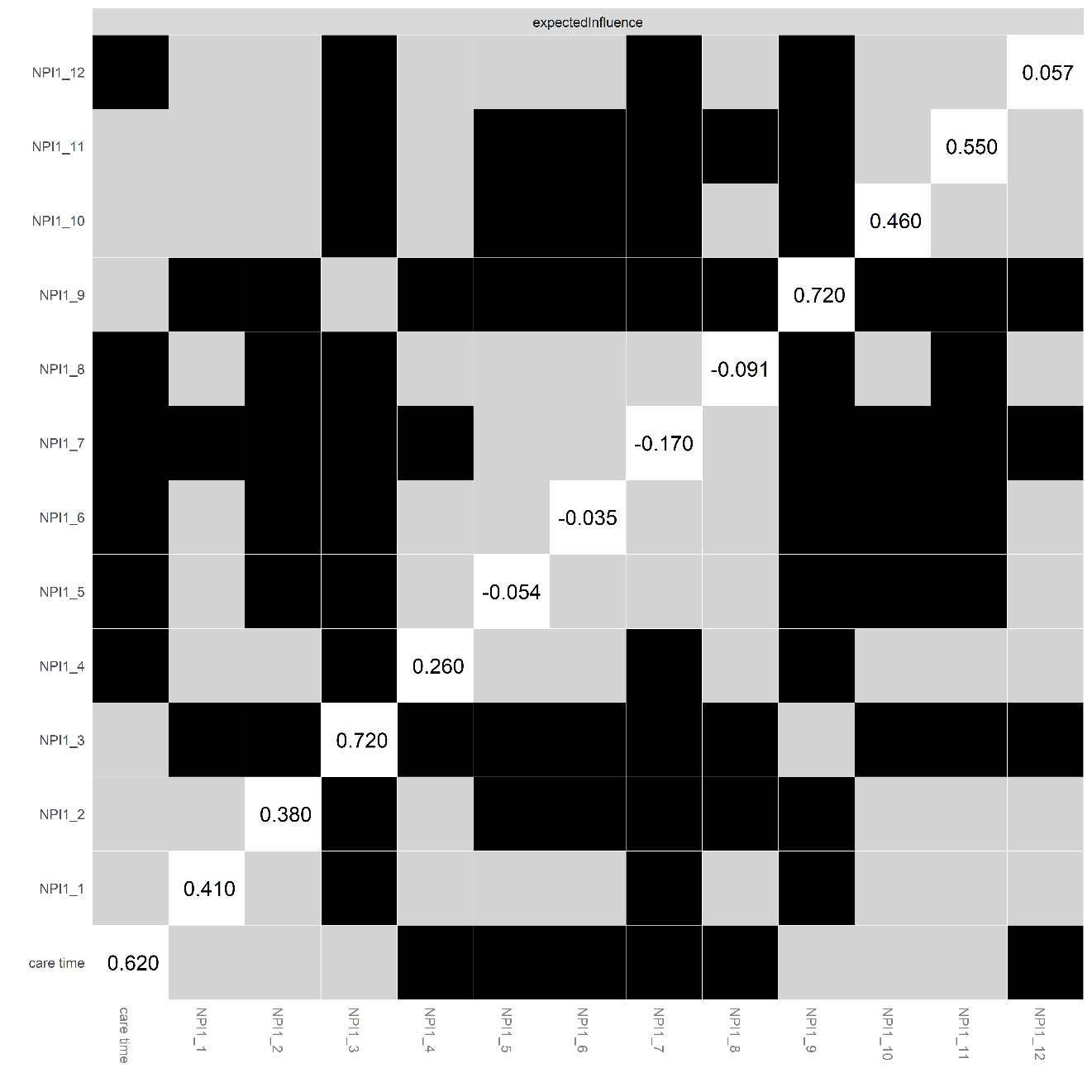


Figure S4. Bootstrapped difference test for expected Influence in the BPSD-related severity and care time network.

Table S2. All edges weights within the BPSD-related distress and care time network

| Variables | care time | NPI2_1 | NPI2_2 | NPI2_3 | NPI2_4 | NPI2_5 | NPI2_6 | NPI2_7 | NPI2_8 | NPI2_9 | NPI2_10 | NPI2_11 | NPI2_12 |
| --- | --- | --- | --- | --- | --- | --- | --- | --- | --- | --- | --- | --- | --- |
| care time | 0.00 |  |  |  |  |  |  |  |  |  |  |  |  |
| NPI2_1 | 0.00 | 0.00 |  |  |  |  |  |  |  |  |  |  |  |
| NPI2_2 | 0.08 | 0.00 | 0.00 |  |  |  |  |  |  |  |  |  |  |
| NPI2_3 | 0.21 | 0.00 | 0.01 | 0.00 |  |  |  |  |  |  |  |  |  |
| NPI2_4 | 0.00 | 0.00 | 0.24 | 0.05 | 0.00 |  |  |  |  |  |  |  |  |
| NPI2_5 | 0.00 | 0.08 | -0.04 | 0.00 | 0.00 | 0.00 |  |  |  |  |  |  |  |
| NPI2_6 | 0.00 | -0.02 | 0.17 | 0.03 | 0.00 | -0.23 | 0.00 |  |  |  |  |  |  |
| NPI2_7 | 0.00 | -0.13 | 0.00 | 0.03 | 0.05 | 0.00 | 0.06 | 0.00 |  |  |  |  |  |
| NPI2_8 | 0.02 | 0.27 | -0.14 | 0.00 | -0.07 | 0.00 | -0.25 | 0.00 | 0.00 |  |  |  |  |
| NPI2_9 | 0.09 | 0.26 | 0.00 | 0.42 | 0.00 | 0.05 | 0.00 | 0.00 | 0.18 | 0.00 |  |  |  |
| NPI2_10 | 0.21 | 0.00 | 0.03 | 0.09 | 0.00 | -0.05 | 0.07 | 0.00 | 0.00 | 0.00 | 0.00 |  |  |
| NPI2_11 | 0.16 | 0.00 | 0.10 | 0.06 | 0.07 | 0.00 | 0.07 | 0.00 | 0.00 | 0.00 | 0.18 | 0.00 |  |
| NPI2_12 | 0.00 | -0.08 | 0.11 | 0.06 | 0.24 | -0.22 | 0.27 | 0.00 | -0.18 | 0.00 | 0.00 | 0.06 | 0.00 |


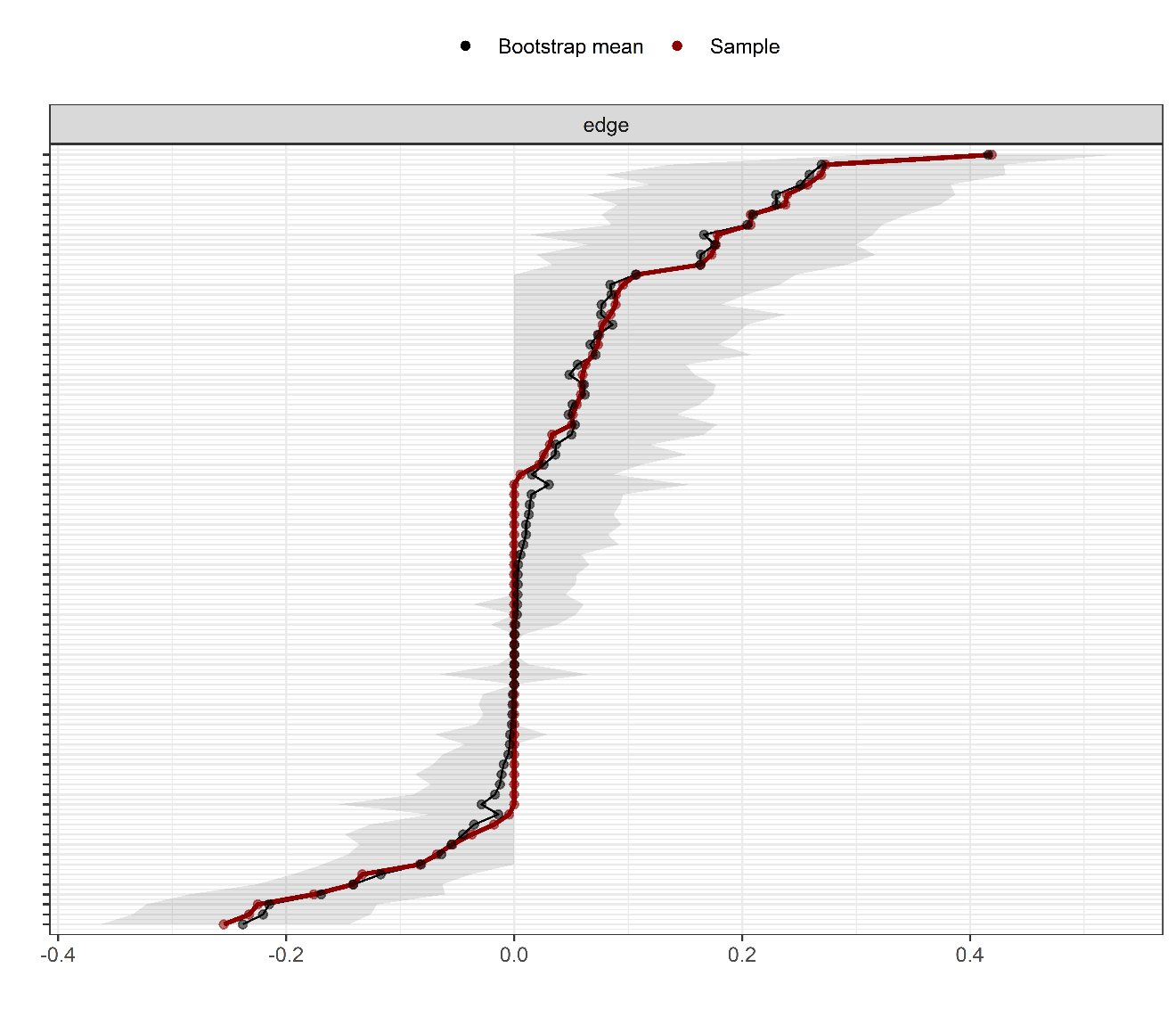


Figure S5. Accuracy of edge weights in the BPSD-related distress and care time network


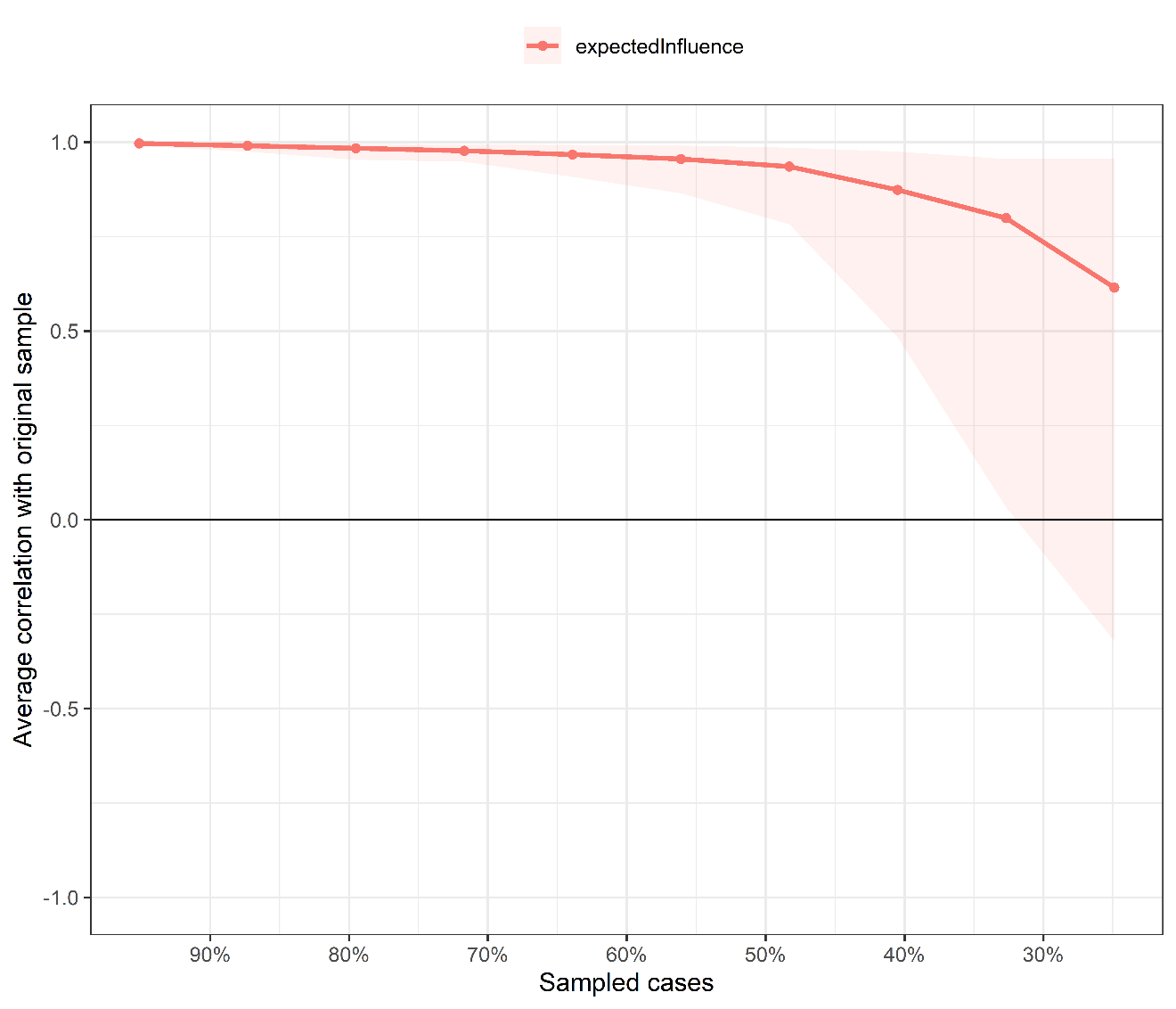


Figure S6. The stability of centrality index-expected influence using case dropping bootstrap in the BPSD-related distress and care time network.


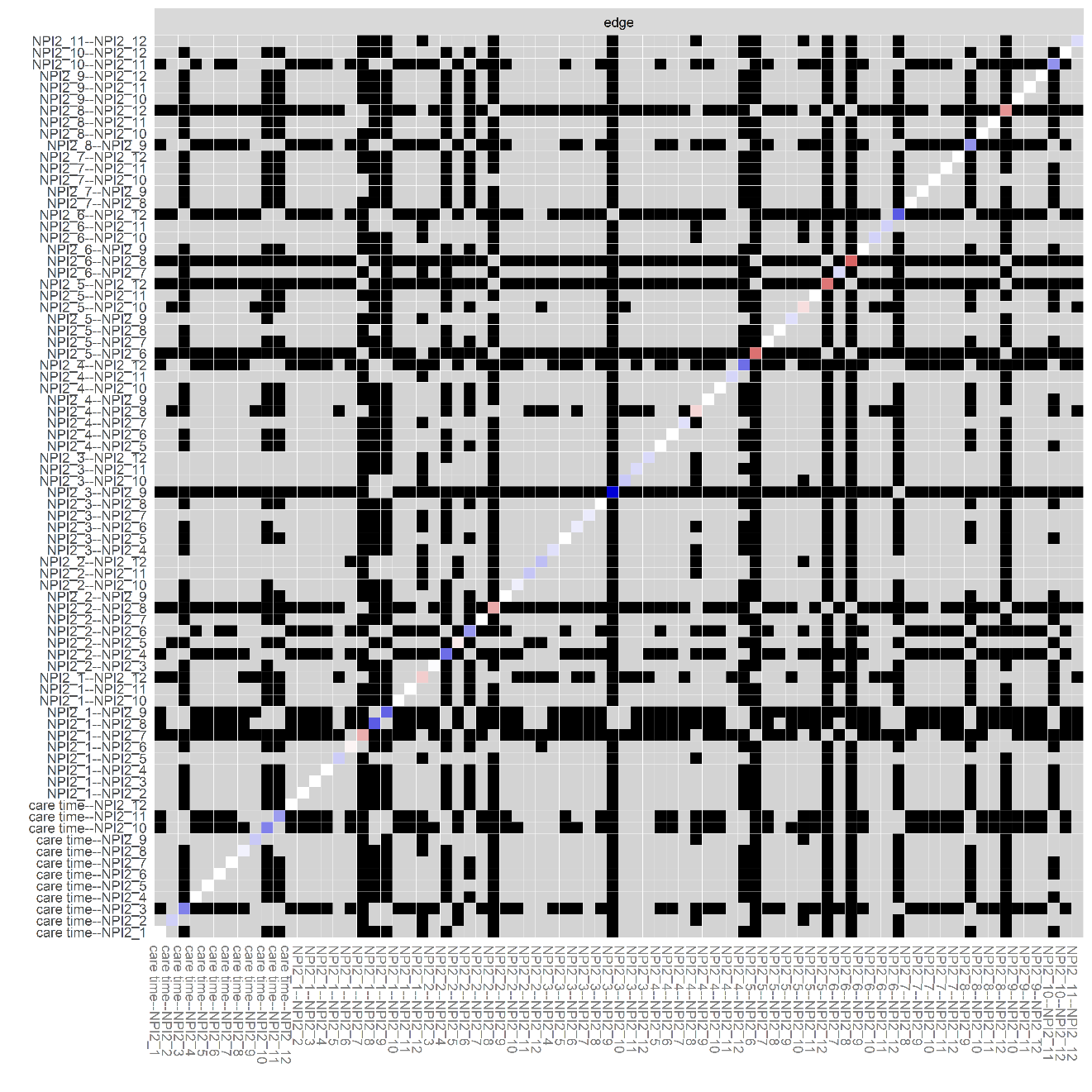


Figure S7. Bootstrapped difference test for edge weights in the BPSD-related distress and care time network


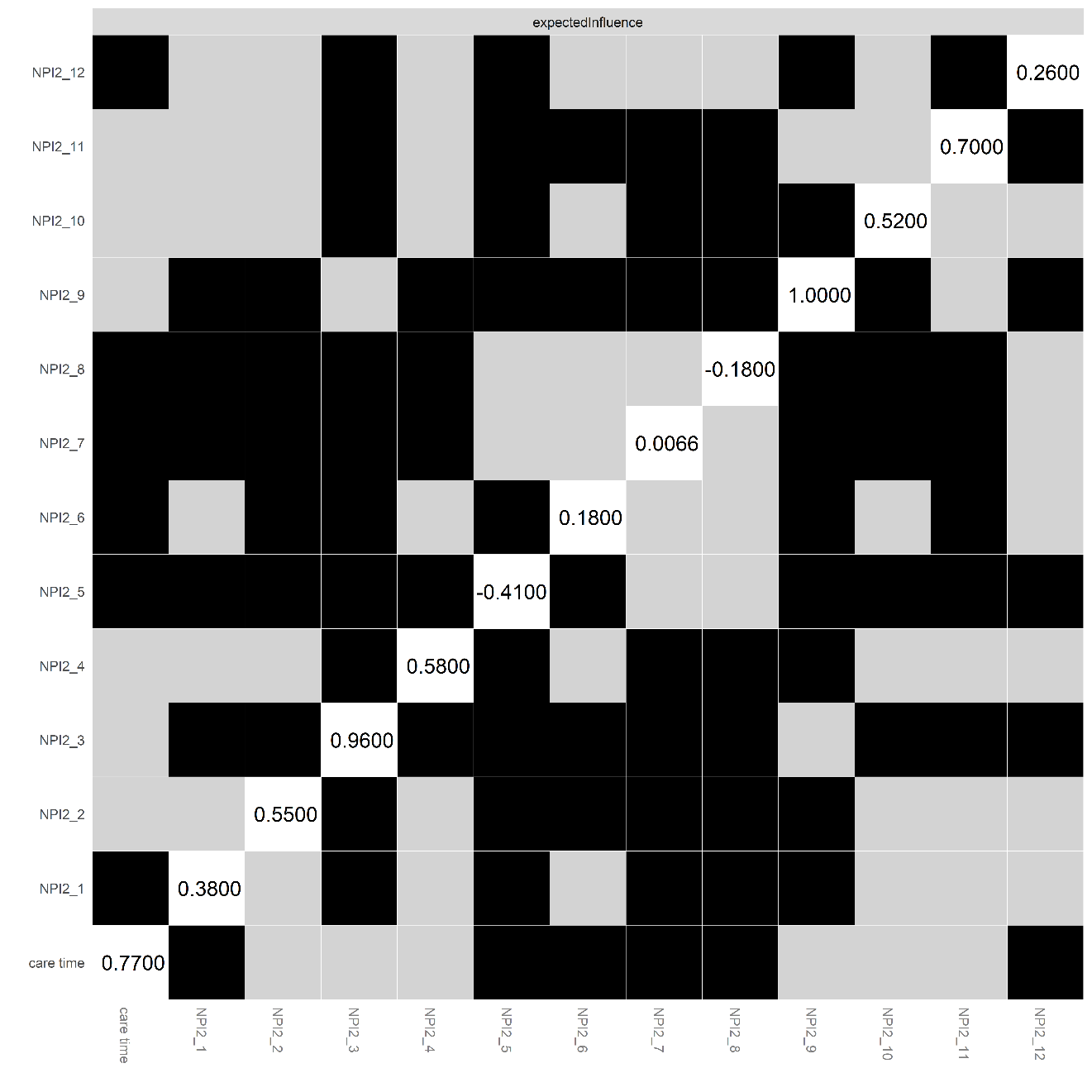


Figure S8. Bootstrapped difference test for expected Influence in the BPSD-related distress and care time network
